# Special Issue “Development and Synthesis of Biologically Active Compounds”

**DOI:** 10.3390/ijms25074015

**Published:** 2024-04-04

**Authors:** Galina A. Gazieva, Konstantin Chegaev

**Affiliations:** 1 N. D. Zelinsky Institute of Organic Chemistry, Russian Academy of Sciences, 47 Leninsky Prosp., 119991 Moscow, Russia; 2 Department of Drug Science and Technology, University of Torino, 10125 Torino, Italy; konstantin.chegaev@unito.it

The intention of this Special Issue is to focus on new achievements in the design, preparation, and in vitro and in vivo biological evaluation of bioactive molecules that can result in the development of natural or artificial potent compounds looking for promising pharmaceuticals and agrochemicals. The relevance of the search for biologically active molecules for the creation of original drugs is due to the widespread occurrence of socially significant diseases such as cardiovascular, oncological, infectious, and neurodegenerative ones.

Cancer is an extremely prevalent disorder responsible for a large number of deaths year by year worldwide [1]. Most of the articles in this Special Issue are devoted to the search for new compounds with antiproliferative and cytotoxic activities and overcoming related problems. Thus, Sodano et al. [2] designed and synthesized a cyclopentadienone conjugated to an inactive dienophile as a new non-metal CO-releasing molecule (CORM), capable of generating CO upon the influence of enzymes. New CORM is toxic to a number of human tumor cell lines including drug-resistant ones. It was efficiently taken up by drug-resistant tumor cells and it was capable of restoring their sensitivity to chemotherapeutic agents by inducing a CO-dependent mitochondrial oxidative stress that resulted in mitochondrial-dependent apoptosis. This work follows on from the authors’ previous works [3,4,5,6,7] showing that certain compounds releasing gaseous signaling molecules, namely nitrogen monoxide (NO) and hydrogen sulfide (H_2_S), can facilitate overcoming the resistance of tumor cells to doxorubicin. These results highlight the significance of small signaling molecule donors in cases of traditional chemotherapy ineffectiveness and thus offer the prospects for novel combination strategies to succeed in chemosensitization and overcoming multidrug resistance.

In screening for bioactivity, there is a problem of the precipitation of a lipophilic compound in an aqueous environment [8]. To overcome the issue, Semenova et al. [9] examined the employment of Pluronics as cosolvents in the in vivo evaluation of some potent microtubule destabilizers. These destabilizers included albendazole, diarylisoxazole [10], and two chalcones [11,12], which were found to be cytotoxic against human cancer cells. The phenotypic sea urchin embryo model was used to provide a rapid and reliable assessment of antiproliferative activity. Phenotypic screening using sea urchin embryo assay demonstrated that all tested compounds preserved antimitotic activity in a water-containing medium when their DMSO or 2-pyrrolidone stock solutions were diluted with Pluronic P123 or Pluronic F127. These results allow one to suggest Pluronics as cosolvents for improving the solubility of lipophilic compounds in water-containing media.

Despite the significant progress of science and technology in the production of synthetic pharmaceuticals, medicines of natural origin play an essential role in cancer therapy. It was evaluated that from 1981 to 2019, about a quarter of all newly approved antitumor drugs were associated with natural compounds. Nevertheless, the development of bioactive compounds of natural origin into drugs has remained challenging due to the difficulties in product isolation and laborious synthesis [13]. These problems are reflected in the Special Issue. Two articles consider cytotoxic naturally occurring compounds and their analogues. Grudzień et al. [14] described the toxicity of xanthohumol and its natural and semisynthetic derivatives using three canine lymphoma and leukemia cell lines. Xanthohumol derivatives induced apoptosis in the studied cell lines. A decrease in Bcl-2 level in cells treated with xanthohumol was revealed by Western blotting. Gettler et al. [15] elaborated a universal synthetic methodology for the enantioselective formation of a benzo[c]oxepine framework found in natural compounds. This approach was successfully applied to the first total synthesis of heterocornol D, earlier extracted from the fungus *Pestalotiopsis heterocornis*. The absolute configurations of the natural heterocornol D stereocenters were determined. In total, synthesis of four stereoisomers of this natural polyketide was realized. The presented synthetic strategy was also extended synthesizing heterocornol C that possessed cytotoxic activity [16]. 

In addition, Cybulski et al. [17] synthesized five new amide conjugates of hydroxycinnamic acids with the known analog of the naturally occurring alkaloid neocryptolepine, i.e., antitumor 5,11-dimethyl-5*H*-indolo[2,3-*b*]quinoline [18,19]. Three conjugates demonstrated significant antiproliferative effect on pancreatic cancer (BxPC-3 and metastatic AsPC-1), breast cancer (MCF-7), and cervical cancer cells (HeLa). One of them showed dose-dependent efficiency towards both BxPC-3 and metastatic AsPC-1 pancreatic cancer cells with a reasonable selectivity index for both tumor cell lines in comparison with normal dermal fibroblasts.

Several publications concern the study of the cytotoxic and antibacterial, antiaggregation, or antiphytopathogenic activities of synthesized compounds. In the investigation by Shybanov et al. [20], the regio- and stereoselective synthesis of novel hydantoin and thiohydantoin-based spiro-compounds was developed. The obtained compounds showed moderate cytotoxicity to MCF7, A549, HEK293T, and VA13 cell lines. Several tested substances had noticeable antibacterial properties against the *Escherichia coli* BW25113 DTC-pDualrep2 strain that has a normal cell wall, but the substances were practically inactive against the *Escherichia coli* BW25113 LPTD-pDualrep2 strain that has corrupted cell walls. 

Castaño et al. [21] prepared several novel series of chalcone–sulfonamide hybrid compounds which were applied for the subsequent construction of the heterocyclic pyrazoline derivatives. The antiproliferative and antituberculosis effects of the obtained compounds were tested. Several compounds exhibited potent antiproliferative effect towards the LOX IMVI (melanoma) cell line and towards the entire group of leukemia cell lines with IC_50_ values ranging from 0.34 to 2.52 μM. Two substances inhibited the growth of *Mycobacterium tuberculosis* H37Rv at concentrations below 10 μM. 

Napiórkowska et al. [22,23,24] devoted several years to the study of the biological properties of benzofuran derivatives, resulting in the identification of bromosubstituted benzofurans possessing high antitumor activity. Among the series of novel 2-acetyl-3-(bromo)methyl benzofurans, two bromomethyl derivatives exhibited selective cytotoxic effect to leukemia cancer cells (K562) compared to that against normal cells (HaCaT) with a favorable therapeutic index. These derivatives induced apoptosis in the explored cancer cells through enhancing the activity of executioner caspases 3/7, had pro-oxidative effect, increased reactive oxygen species, and inhibited the excretion of pro-inflammatory interleukin 6 in K562 cells. The screening for antibacterial activity of benzofurans using standard and clinical strains showed that one compound exhibited a modest effect against Gram-positive strains.

Bogdanov et al. [25] prepared two series of new fluorinated N-benzylisatins and water-soluble isatin-3-hydrazones. Fluorinated N-benzylisatins possessed cytotoxic action against M-HeLa and HuTu 80 cancer-derived cells, inducing apoptosis through mitochondrial membrane dissipation and reactive oxygen species generation in cancer cells. In addition, two compounds exhibited antiplatelet effect at the reference compound, acetylsalicylic acid, level. Among the new water-soluble pyridinium isatin-3-acylhydrazones, three compounds exhibited the most potent activity towards fungal and bacterial phytopathogens and can be employed for developing agrochemicals to control plant diseases.

To continue a previous investigation [26], Vinogradova et al. [27] evaluated fungicidal activity of new S-alkyl substituted thioglycolurils against six phytopathogenic fungi and two pathogenic yeasts. Several S-alkyl thioglycolurils inhibited the growth of *Venturia inaequalis* and *Rhizoctonia solani* mycelium by 85–100% with somewhat lower activity towards other phytopathogens. Ethylsulfanyl-substituted compounds demonstrated significant activity towards yeast *Candida albicans*. For these compounds, their hemolytic effect and cytotoxicity were assessed in relation to human red blood cells and human embryonic kidney cells, respectively. Two ethylsulfanyl derivatives demonstrated low hemolytic and cytotoxic activities in connection with normal human cells along with strong antifungal effect towards *Candida albicans*.

Aksenov et al. [28] synthesized diverse 3,5-di-(hetero)aryl-4-benzyl-substituted α,β-unsaturated γ-hydroxy butyrolactams. These compounds are in great demand in synthetic organic and medicinal chemistry [29]. 

Human lactate dehydrogenase (hLDH), in particular isoform hLDHA, has been identified as a therapeutic target for treating various diseases such as cancer [30], vascular diseases [31,32], inflammatory diseases [33], and primary hyperoxaluria [34]. Salido et al. [35] synthesized the analogues of naturally occurring A-type proanthocyanidin containing a 2,8-dioxabicyclo[3.3.1]nonane core. Nine 2,8-dioxabicyclo[3.3.1]nonane derivatives were characterized by IC_50_ values of less than 10 µM towards hLDHA. The enantiomers of the most effective and selective substances were separated using chiral HPLC. The study of inhibitory activity of pure enantiomers derived from 2,8-dioxabicyclo[3.3.1]nonane showed that the absolute configuration had little effect on hLDHA inhibition. The authors ascertained that dextrorotatory enantiomers are preferable because the kinetic investigation of hLDHA inhibition by enantiomers manifested a noncompetitive inhibition behavior for both enantiomers, with the dextrorotatory enantiomer being 2–4 times more effective in inhibiting the hLDHA enzyme.

Catalytic antibodies possess numerous functions compared to monoclonal antibodies due to their specific ability to decompose antigens enzymatically. Hence, a plethora of such antibodies and their subunits have been prepared since 1989 [36,37,38,39,40]. Nonaka et al. [41] performed methodical research on two catalytic antibody light chains, #7TR and H34, by modifying the purification techniques, pH values, and reagents used. In the peptidase activity tests and kinetic studies, high catalytic activity of the light chains took place when the chains were obtained under basic conditions. These results indicate that a slight structure adjustment of the catalytic antibodies occurs during the purification procedure to improve the catalytic activity whereas the antigen recognition capability remains unchanged. When the #7TR and H34 chains were produced under the reported conditions, they highly increased the degradability of Amyloid-beta and PD-1 peptides, respectively. These results offer exciting prospects of using catalytic antibodies to alter the course of Alzheimer’s disease and cancer. 

Human immunodeficiency virus (HIV) causes one of the deadliest disorders, acquired immunodeficiency syndrome (AIDS) [42,43]. At present, one of the intensively evolving fields of organic and medicinal chemistry is the design and discovery of new substances that can inhibit one of the HIV enzymes, particularly HIV-1 integrase [44,45]. One review [46] presented an assay of the publications concerning the synthetic approaches to obtain pyridine-comprising HIV-1 integrase inhibitors from 2003 to the present. The review emphasizes that the area of chemistry related to the preparation of new HIV-1 integrase inhibitors comprising pyridine core remains in demand and is needed for the development and search for new medicines against HIV.

## References

[1] Chhikara B.S., Parang K. (2023). Global Cancer Statistics 2022: The trends projection analysis. Chem. Biol. Lett..

[2] Sodano F., Rolando B., Lazzarato L., Costamagna C., Failla M., Riganti C., Chegaev K. (2023). Use of Enzymatically Activated Carbon Monoxide Donors for Sensitizing Drug-Resistant Tumor Cells. Int. J. Mol. Sci..

[3] Chegaev K., Riganti C., Lazzarato L., Rolando B., Guglielmo S., Campia I., Fruttero R., Bosia A., Gasco A. (2011). Nitric oxide donor doxorubicins accumulate into doxorubicin-resistant human colon cancer cells inducing cytotoxicity. ACS Med. Chem. Lett..

[4] Riganti C., Miraglia E., Viarisio D., Costamagna C., Pescarmona G., Ghigo D., Bosia A. (2005). Nitric oxide reverts the resistance to doxorubicin in human colon cancer cells by inhibiting the drug efflux. Cancer Res..

[5] Masetto F., Chegaev K., Gazzano E., Mullappilly N., Rolando B., Arpicco S., Fruttero R., Riganti C., Donadelli M. (2020). MRP5 nitration by NO-releasing gemcitabine encapsulated in liposomes confers sensitivity in chemoresistant pancreatic adenocarcinoma cells. Biochim. Biophys. Acta Mol. Cell Res..

[6] Chegaev K., Rolando B., Cortese D., Gazzano E., Buondonno I., Lazzarato L., Fanelli M., Hattinger C.M., Serra M., Riganti C. (2016). H_2_S-Donating Doxorubicins May Overcome Cardiotoxicity and Multidrug Resistance. J. Med. Chem..

[7] Buondonno I., Gazzano E., Tavanti E., Chegaev K., Kopecka J., Fanelli M., Rolando B., Fruttero R., Gasco A., Hattinger C. (2019). Endoplasmic reticulum-targeting doxorubicin: A new tool effective against doxorubicin-resistant osteosarcoma. Cell. Mol. Life Sci..

[8] Popa-Burke I.G., Issakova O., Arroway J.D., Bernasconi P., Chen M., Coudurier L., Galasinski S., Jadhav A.P., Janzen W.P., Lagasca D. (2004). Streamlined system for purifying and quantifying a diverse library of compounds and the effect of compound concentration measurements on the accurate interpretation of biological assay results. Anal. Chem..

[9] Semenova M.N., Melik-Nubarov N.S., Semenov V.V. (2023). Application of Pluronics for Enhancing Aqueous Solubility of Lipophilic Microtubule Destabilizing Compounds on the Sea Urchin Embryo Model. Int. J. Mol. Sci..

[10] Semenova M.N., Demchuk D.V., Tsyganov D.V., Chernysheva N.B., Samet A.V., Silyanova E.A., Kislyi V.P., Maksimenko A.S., Varakutin A.E., Konyushkin L.D. (2018). Sea urchin embryo model as a reliable in vivo phenotypic screen to characterize selective antimitotic molecules. Comparative evaluation of combretapyrazoles, -isoxazoles, -1,2,3-triazoles, and -pyrroles as tubulin-binding agents. ACS Comb. Sci..

[11] Semenov V.V., Semenova M.N. (2015). Polyalkoxyflavonoids as inhibitors of cell division. Russ. Chem. Rev..

[12] Ducki S., Rennison D., Woo M., Kendall A., Chabert J.F., McGown A.T., Lawrence N.J. (2009). Combretastatin-like chalcones as inhibitors of microtubule polymerization. Part 1: Synthesis and biological evaluation of antivascular activity. Bioorg. Med. Chem..

[13] Huang M., Lu J.J., Ding J. (2021). Natural Products in Cancer Therapy: Past, Present and Future. Nat. Prod. Bioprospect..

[14] Grudzień M., Pawlak A., Tronina T., Kutkowska J., Kruszyńska A., Popłoński J., Huszcza E., Rapak A. (2023). The Effect of Xanthohumol Derivatives on Apoptosis Induction in Canine Lymphoma and Leukemia Cell Lines. Int. J. Mol. Sci..

[15] Gettler J., Čarný T., Markovič M., Koóš P., Samoľová E., Moncoľ J., Gracza T. (2023). Synthetic Study of Natural Metabolites Containing a Benzo[*c*]oxepine Skeleton: Heterocornol C and D. Int. J. Mol. Sci..

[16] Lei H., Lin X., Han L., Ma J., Dong K., Wang X., Zhong J., Mu Y., Liu Y., Huang X. (2017). Polyketide derivatives from a marine-sponge-associated fungus *Pestalotiopsis heterocornins*. Phytochemistry.

[17] Cybulski M., Sidoryk K., Zaremba-Czogalla M., Trzaskowski B., Kubiszewski M., Tobiasz J., Jaromin A., Michalak O. (2024). The Conjugates of Indolo[2,3-*b*]quinoline as Anti-Pancreatic Cancer Agents: Design, Synthesis, Molecular Docking and Biological Evaluations. Int. J. Mol. Sci..

[18] Peczyńska-Czoch W., Pognan F., Kaczmarek Ł., Boratyński J. (1994). Synthesis and structure-activity relationship of methylsubstituted indolo[2,3-b]quinolines: Novel cytotoxic, DNA topoisomerase II inhibitors. J. Med. Chem..

[19] Kaczmarek Ł., Peczyńska-Czoch W., Osiadacz J., Mordarski M., Sokalski W.A., Boratyński J., Marcinkowska E., Glazman-Kuśnierczyk H., Radzikowski C. (1999). Synthesis, and cytotoxic activity of some novel indolo[2,3-b]quinoline derivatives: DNA topoisomerase II inhibitors. Bioorg. Med. Chem..

[20] Shybanov D.E., Kukushkin M.E., Hrytseniuk Y.S., Grishin Y.K., Roznyatovsky V.A., Tafeenko V.A., Skvortsov D.A., Zyk N.V., Beloglazkina E.K. (2023). [4+2]-Cycloaddition to 5-Methylidene-Hydantoins and 5-Methylidene-2-Thiohydantoins in the Synthesis of Spiro-2-Chalcogenimidazolones. Int. J. Mol. Sci..

[21] Castaño L.F., Quiroga J., Abonia R., Insuasty D., Vidal O.M., Seña R., Rubio V., Puerto G., Nogueras M., Cobo J. (2022). Synthesis, Anticancer and Antitubercular Properties of New Chalcones and Their Nitrogen-Containing Five-Membered Heterocyclic Hybrids Bearing Sulfonamide Moiety. Int. J. Mol. Sci..

[22] Napiórkowska M., Kumaravel P., Amboo Mahentheran M., Kiernozek-Kalińska E., Grosicka-Maciąg E. (2024). New Derivatives of 1-(3-Methyl-1-Benzofuran-2-yl)Ethan-1-one: Synthesis and Preliminary Studies of Biological Activity. Int. J. Mol. Sci..

[23] Napiórkowska M., Cieślak M., Kaźmierczak-Barańska J., Królewska-Golińska K., Nawrot B. (2019). Synthesis of new derivatives of benzofuran as potential anticancer agents. Molecules.

[24] Krolewska-Golinska K., Cieslak M.J., Sobczak M., Dolot R., Radzikowska-Cieciura E., Napiorkowska M., Wybranska I., Nawrot B. (2019). Novel Benzo[B]Furans with Anti-Microtubule Activity Upregulate Expression of Apoptotic Genes and Arrest Leukemia Cells in G2/M Phase. Anti-Cancer Agents Med. Chem..

[25] Bogdanov A.V., Neganova M., Voloshina A., Lyubina A., Amerhanova S., Litvinov I.A., Tsivileva O., Akylbekov N., Zhapparbergenov R., Valiullina Z. (2023). Anticancer and Antiphytopathogenic Activity of Fluorinated Isatins and Their Water-Soluble Hydrazone Derivatives. Int. J. Mol. Sci..

[26] Gazieva G.A., Anikina L.V., Nechaeva T.V., Pukhov S.A., Karpova T.B., Popkov S.V., Nelyubina Y.V., Kolotyrkina N.G., Kravchenko A.N. (2017). Synthesis and biological evaluation of new substituted thioglycolurils, their analogues and derivatives. Eur. J. Med. Chem..

[27] Vinogradova E.E., Alekseenko A.L., Popkov S.V., Kolotyrkina N.G., Kravchenko A.N., Gazieva G.A. (2023). Synthesis and Evaluation on the Fungicidal Activity of S-Alkyl Substituted Thioglycolurils. Int. J. Mol. Sci..

[28] Aksenov A.V., Aksenov D.A., Kurenkov I.A., Leontiev A.V., Aksenov N.A. (2023). A New, Convenient Way to Fully Substituted α,β-Unsaturated γ-Hydroxy Butyrolactams. Int. J. Mol. Sci..

[29] Nay B., Riache N., Evanno L. (2009). Chemistry and Biology of Non-Tetramic γ-Hydroxy-γ-Lactams and γ-Alkylidene-γ-Lactams from Natural Sources. Nat. Prod. Rep..

[30] Doherty J.R., Cleveland J.L. (2013). Targeting lactate metabolism for cancer therapeutics. J. Clin. Investig..

[31] Fraile-Martinez O., García-Montero C., Álvarez-Mon M.A., Gomez-Lahoz A.M., Monserrat J., Llavero-Valero M., Ruiz-Grande F., Coca S., Alvarez-Mon M., Buján J. (2022). Venous wall of patients with chronic venous disease exhibits a glycolytic phenotype. J. Pers. Med..

[32] Kim J.-H., Bae K.-H., Byun J.-K., Lee S., Kim J.-G., Lee I.K., Jung G.-S., Lee Y.M., Park K.G. (2017). Lactate dehydrogenase-A is indispensable for vascular smooth muscle cell proliferation and migration. Biochem. Biophys. Res. Commun..

[33] Gupta G.S. (2022). The lactate and the lactate dehydrogenase in inflammatory diseases and major risk factors in COVID-19 patients. Inflammation.

[34] Martín-Higueras C., Torres A., Salido E. (2017). Molecular therapy of primary hyperoxaluria. J. Inherit. Metab. Dis..

[35] Salido S., Alejo-Armijo A., Altarejos J. (2023). Synthesis and *h*LDH Inhibitory Activity of Analogues to Natural Products with 2,8-Dioxabicyclo[3.3.1]nonane Scaffold. Int. J. Mol. Sci..

[36] Paul S., Volle D.J., Beach C.M., Johnson D.R., Powell M.J., Massey R.J. (1989). Catalytic Hydrolysis of vasoactive intestinal peptide by human autoantibody. Science.

[37] Shuster A.M., Gololobov G.V., Kvashuk O.A., Bogomolova A.E., Smirnov I.V., Gabibov A.G. (1992). DNA hydrolyzing autoantibodies. Science.

[38] Lacroix-Desmazes S., Sooryanarayana M.A., Bonnemain C., Stieltjes N., Pashov A., Sultan Y., Hoebeke J., Kazatchkine M.D., Kaveri S.V. (1999). Catalytic activity of antibodies against factor VIII in patients with hemophilia A. Nat. Med..

[39] Parkhomenko T.A., Buneva V.N., Tyshkevich O.B., Generalov I.I., Doronin B.M., Nevinsky G. (2010). DNA-hydrolyzing activity of IgG antibodies from the sera of patients with tick-borne encephalitis. Biochimie.

[40] Tolmacheva A.S., Nevinsky G.A. (2022). Essential Protective Role of Catalytically Active Antibodies (Abzymes) with Redox Antioxidant Functions in Animals and Humans. Int. J. Mol. Sci..

[41] Nonaka T., Taguchi H., Uda T., Hifumi E. (2022). Obtaining Highly Active Catalytic Antibodies Capable of Enzymatically Cleaving Antigens. Int. J. Mol. Sci..

[42] Deeks S., Overbaugh J., Phillips A., Buchbinder S. (2015). HIV infection. Nat. Rev. Dis. Primers.

[43] Bekker L.-G., Beyrer C., Mgodi N., Lewin S.R., Delany-Moretlwe S., Taiwo B., Masters M.C., Lazarus J.V. (2023). HIV infection. Nat. Rev. Dis. Primers.

[44] Scarci K.K., Havens J.P., Podany A.T., Avedissian S.N., Fletcher C.V. (2020). HIV-1 Integrase Inhibitors: A Comparative Review of Efficacy and Safety. Drugs.

[45] Sun J., Kessl J.J. (2024). Optimizing the Multimerization Properties of Quinoline-Based Allosteric HIV-1 Integrase Inhibitors. Viruses.

[46] Starosotnikov A.M., Bastrakov M.A. (2023). Recent Developments in the Synthesis of HIV-1 Integrase Strand Transfer Inhibitors Incorporating Pyridine Moiety. Int. J. Mol. Sci..

